# Environmental Exposure to Emissions from Petrochemical Sites and Lung Cancer: The Lower Mississippi Interagency Cancer Study

**DOI:** 10.1155/2010/759645

**Published:** 2010-03-14

**Authors:** Neal Simonsen, Richard Scribner, L. Joseph Su, Donna Williams, Brian Luckett, Tong Yang, Elizabeth T. H. Fontham

**Affiliations:** ^1^School of Public Health, Louisiana State University Health Sciences Center, Suite 1400, 1615 Poydras St, New Orleans, LA 70112, USA; ^2^National Cancer Institute, Rockville, MA 20892, USA; ^3^School of Medicine, Louisiana State University Health Sciences Center, New Orleans, LA 70112, USA

## Abstract

To investigate potential links between environmental exposure to petrochemical plant emissions and lung cancer, a population-based case-control study (LMRICS) was conducted in eleven Louisiana parishes bordering the Mississippi River. Cases and age, gender, and race-matched controls were interviewed regarding potential risk factors. Residential history was geocoded to provide indices of long-term proximity to industrial sites. Cases were more likely to have lived near a petrochemical site. Models adjusted for other risk factors, however, showed small or no association with lung cancer (odds ratio for residence within a half-mile of a site = 1.10, 95% confidence interval 0.58–2.08). While associations were strongest for exposures exceeding 15 years, none approached statistical significance and there was no clear dose-response across exposure duration, distance categories, or when sites were grouped according to carcinogenicity rating of chemical releases. Residential proximity to petrochemical plants along the lower Mississippi thus showed no significant association with lung cancer.

## 1. Introduction

The petrochemical industry in Louisiana includes over 100 plants. Together they account for a quarter of the total U.S. petrochemical production, ranking second only to Texas in total refinery output [1]. Most of these facilities are located along the Mississippi River in Southeast Louisiana, leading to its identification as the state's industrial corridor. 

The state of Louisiana has consistently presented higher rates of lung cancer incidence and mortality compared to the U.S. as a whole for the past five decades. During 1998–2003, for example, white male lung and bronchial cancer incidence per 100,000 persons was 108.8 in Louisiana compared to 75.3 for the U.S. [2]. Spurred in part by the conjunction of industry and lung cancer, concerns have arisen that a petrochemical pollution-driven “cancer corridor” could exist along the lower Mississippi. 

A review by Whitrow et al. [3] identified ten case-control and four cohort studies done between 1982 and 2003 that measured environmental factors and assessed their association with lung cancer while attempting to address both tobacco smoke and occupation in their analyses. Most studies made use of general indices of air pollution rather than measures of specific carcinogens. Overall, while 8 out of 14 studies found a significant association between one or more exposure indices and lung cancer, only five evaluated evidence of a dose-response relationship, with two finding a significant positive trend, one a positive trend only among smokers, one no significant trend, and one a significant negative trend. Most studies were able to address only a limited number of potential risk factors, emphasizing the need for comprehensive individual-level risk factor quantification in future studies. 

A number of studies of varying designs have investigated proximity to a specific industrial site(s). Most focused on nonferrous smelters, with more finding a significant association between smelter proximity and lung cancer [4–7] than not [8, 9]. Kaldor et al. [10] divided a California county into four regions of increasing estimated petrochemical plant emissions based on hydrocarbon and SO_2_ monitoring data from 1975. Lung cancer rates for census tract groupings rose significantly with increasing regional exposure among white males, but not among white females. Although the analyses considered socioeconomic status indices of the tract groups, smoking was not addressed. Sans et al. [11] evaluated cancer rates around a petrochemical plant in South Wales. Using mortality data from 1981–1991, deaths were mapped to within 3 or 7.5 km of the plant, and mortality observed in these areas was compared to that expected based on rates for England and Wales as a whole. No significant increase in mortality was found, although observed mortality exceeded expected at 0–3 km. Age, gender, a social deprivation index, and region were adjusted for based on census information for the tract of residence, but no adjustment was made for smoking. 

One older and two recent studies examined associations between cancer and the petrochemical industry or industrial corridor in Louisiana. Gottlieb et al. [12] abstracted cause of death, usual residence, and occupation from death certificates for 20 Louisiana parishes during 1960–1975. Length of residence was obtained from public records. A total of 1418 person dying from lung cancer were matched with 1429 controls dying from causes other than cancer on age, gender, parish, and year of death. The odds of lung cancer were compared for persons living within less than one mile and those living within 1 to three miles of a specific type of industry. Higher odds ratios were observed for those living within one mile of either a chemical or a petroleum industry site for at least ten years (OR = 1.51 and 1.65, respectively). Neither of these differences reached statistical significance, with the number of exposed cases never exceeding 53. A significantly elevated OR appeared only for subanalyses restricted to decedents whose usual industry of occupation was judged of low risk for lung cancer, and then only for proximity to chemical industry sites with at least 100 employees. The reliance on information from death certificates precluded control for smoking or additional risk factors. 

A study of the Norco Manufacturing Complex, a large refining and manufacturing complex, followed workers from 1973 through 1999 and found no increase in mortality from respiratory cancer or other major causes of death [13]. The standardized mortality ratio (SMR) for respiratory cancer was in fact significantly lower for workers compared to the general population of the state or region. A lack of information on smoking habits makes this difference difficult to interpret. It also addressed only occupational rather than environmental exposure. 

Tsai et al. [14] carried out an ecologic study to compare age-adjusted mortality rates in Louisiana overall and in just the industrial corridor with those in the U.S. for each ten-year period from 1970 through 1999. While white male lung cancer death rates were consistently higher for Louisiana than for the U.S. as a whole, rates in the corridor were lower than those in the state as a whole, and this difference was statistically significant for the first and third decades under consideration. A similar pattern of association was seen for nonwhite males, but the differences between the corridor and the state were much smaller. No consistent difference occurred for women. While these results do not support an increased lung cancer risk with residence in the industrial corridor, it is notable that the corridor population displayed lower poverty and unemployment along with higher income and educational levels than the state as a whole, factors likely to lower expected death rates. The results were not adjusted for smoking habits. 

In order to further investigate the potential link between environmental exposure to petroleum and chemical plant emissions and lung cancer, a case-control study was carried out as part of the Lower Mississippi River Interagency Cancer Study (LMRICS). This case-control study was designed to incorporate detailed information on proximity to key industrial sites as well as information allowing control for other known and potential individual-level risk factors including smoking habits and occupational history. The basic hypothesis understudy was that residential proximity to petrochemical industry sites, particularly those releasing hydrocarbons rated as suspected human carcinogens, is associated with lung cancer risk in the lower Mississippi River industrial corridor.

## 2. Materials and Methods

**Figure 1:**
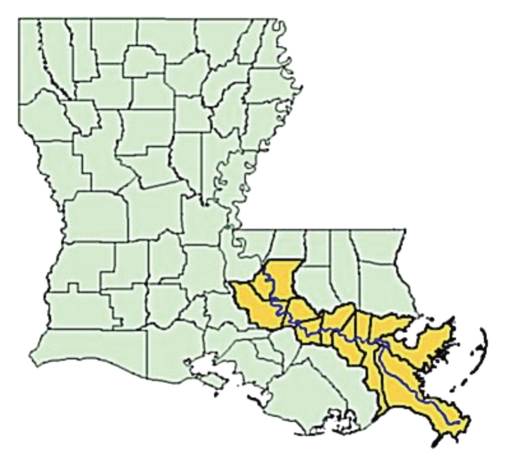
Louisiana parishes included in the LMRICS case-control study (Study parishes are shown in yellow.).

The LMRICS study-area included 11 Louisiana parishes (counties) bordering the Mississippi River: East Baton Rouge, West Baton Rouge, Iberville, Ascension, St. John, St. James, St. Charles, Orleans, Jefferson, St. Bernard, and Plaquemine parishes (Figure 1). This incorporated two major metropolitan areas, Baton Rouge and New Orleans.

A rapid case ascertainment system encompassing 25 medical institutions was set up within the catchment area to identify persons newly diagnosed with lung cancer. Ascertainment was carried out in conjunction with the Louisiana Tumor Registry to assure completeness. Eligible cases were those aged 20–74 years and residing in one of the LMRICS parishes at the time of diagnosis with histologically confirmed primary carcinoma of the lung (International Classification of Diseases-9, 162.2–162.9), diagnosed between January 1, 1998 and March 1, 2001, and with no prior history of cancer (except basal or squamous carcinoma of the skin). Further, only persons living and able to participate in an interview were eligible for the study; no proxy respondents utilized. Controls were identified from state driver's license and personal identification card files and frequency matched to cases on age, gender, and race using stratified random sampling of residents in the study parishes. Determination of race was based on self-report, obtained first from medical records and confirmed by the respondent at interview. While no exclusions were made based on race or ethnicity, most study subjects were either African-American or non-Hispanic Caucasian. The study was approved by the Institutional Review Board of the Louisiana State University Health Sciences Center and adhered to all applicable protocols of other participating medical institutions. All study volunteers gave their informed consent before inclusion in this study.

Participants undertook an extensive interview using a standardized questionnaire to identify potential risk factors. In addition to demographics, lifetime history of cigarette and other tobacco usage was obtained along with estimates of environmental tobacco smoke exposure. Residential histories were elicited for every place since 1970 where the subject resided for at least six months. All jobs held for at least six months were recorded, along with self-reported exposure to any of 12 potential lung carcinogens on each job. Vitamin intake, diet, family, and medical histories were also obtained. In addition, blood and/or buccal cell samples were obtained from participants, and tumor tissue blocks were obtained from participating hospitals as available.

In order to determine residential proximity to industrial sites, every reported address of residence held by participants from 1970 through 1997 inclusive was geocoded using the Map Marker geocoding engine. Residence data were missing for less than 2% of the study period on average. Exact address matches were obtained for 95% of the reported residential addresses. Some residences could only be traced to a specific street and block. When this occurred, the address was mapped to the midpoint of the street. Other addresses were limited to rural route box numbers, which were mapped to the centroid of the zip code boundary for that box. This extended geocoding to 97% of the residential addresses. Residences with missing or unlocatable addresses were not mapped and thus were not included in subsequent analyses. Following the primary analyses, sensitivity analyses were carried out to assure that the exclusion of residences geocoded to zip code centroids did not measurably affect the results.

Industrial sites with potential for toxic chemical emissions to the environment in the LMRICS study area were identified in conjunction with the state's Office of Public Health. The boundaries of each of these sites were determined from aerial or satellite photographs, verified with site representatives, and mapped, again using Map Marker. Sites were characterized in three ways. First, all sites were considered as a whole, without regard to specific emissions. Second, sites were classified on the basis of their Standard Industrial Classification (SIC) code as either belonging to the petrochemical industry or not. The specific SIC codes used to identify petrochemical sites are provided in the appendix. Third, sites were classified on the basis of the International Agency for Research on Cancer (IARC) carcinogen rating assigned to their specific chemical releases [15]. To generate this classification, U.S. EPA mandated Toxic Release Inventory (TRI) data compiled by the state's Office of Public Health was obtained for every site from 1989 through 1999. Focusing on releases to air as the most relevant route of exposure for lung cancer, every chemical reported to have been released for each year of the site's active operation was identified. Among those chemicals, the chemical rated by IARC with the strongest evidence as a human carcinogen was determined and the IARC carcinogen rating of that chemical was then assigned to the site. In this way, sites were classified as either 1 (human carcinogen emitting), 2A (probable human carcinogen emitting), 2B (possible human carcinogen emitting), or none of the preceding (i.e., no reported releases of chemicals identified as at least possible human carcinogens by IARC).

To determine an individual's proximity to industrial sites, the distance from that individual's residence to the closest boundary of each site was then determined. Using this measure, proximity was then characterized in terms of whether an individual's residence fell within the area extending outward from the boundaries of a site to a given distance, or “buffer.” Three such buffers were computed: 0.5 miles, 1 mile, and 2 miles. Distances were considered both on the basis of whether the individual was ever within a particular distance in the course of their residential history from 1970 to 1997 and on the basis of how many years over the course of their residential history they were within a particular distance of a given site type.

For purposes of the primary analyses, site distance calculations were restricted to only those years within which a particular site was actually active. For years before (or after) a site was actually operational, distances were set to missing. For computational reasons, all distances exceeding 30,000 m were set to 30,000 on the assumption that exposure potential at such distances was essentially null. This had no effect on the primary analyses, which were based on residence within buffers of up to 2 miles (3,219 m) in radius. Analyses were carried out both with and without a 5-year lag period to reflect the assumption that past exposures are more important than very recent ones. Incorporating a more lengthy lag period such as 15 or 20 years in the main analyses would lose over half of the residential (and hence exposure) history available for the study and would ignore potential postinitiation promotional effects by those exposures. Exploratory analyses to assess the sensitivity of results to a 15-year lag were nevertheless carried out.

Following simple descriptive statistics and comparisons of means, odds ratios were computed using SAS version 9.1 software. Logistic regression analyses conditioned on age (20–39, 40–54, 55–64, and 65–74 years), gender, and race were run in order to retain the frequency matching incorporated in the study's sampling scheme (SAS 9.1's PROC PHREG). Unconditional logistic regression analyses incorporating binary indicators for gender and race (Caucasian non-Hispanic) as well as a continuous age term were then carried out (PROC LOGISTIC). All adjusted models also included current smoking status, duration and intensity of smoking, and educational level. Unconditional logistic regression results are presented here as they differed little from the conditional regression results.

## 3. Results and Discussion

### 3.1. Study Enrollment and Descriptive Comparisons

A total of 998 potentially eligible lung cancer cases were ascertained during the study period. Of these, 119 (12%) refused to participate. A further 54 (5%) were uncontactable, while 363 were deceased, or deemed too ill to interview. The remaining 462 cases comprised the participating study sample. Of 767 apparently eligible controls, 442 (58%) were contacted and agreed to participate. This yields a response rate among eligible cases of 73% and 58% among controls. Since eligibility could not be fully assessed among nonrespondents, the pool of true eligibles is overestimated. Nonrespondents tended to be slightly older than respondents among cases and younger (by over four years) among controls. For the main analytical dataset, seven cases and five controls that were missing values for one or more of the variables used in the fully adjusted logistic regression were excluded to ensure comparability of the populations used in all comparisons, leaving a total of 455 cases and 437 controls. 

Table 1 presents the basic characteristics of the study population. The study population consisted primarily of white males, with a mean age of 60 years. As expected, the frequency-matched cases and controls showed little difference in age, gender, or race. Controls averaged slightly more residences during the study period than cases, and nearly all (98%) of these residences were successfully geocoded. Cases were much more likely to be smokers, with a significantly higher average duration and intensity of tobacco smoking than controls.

Basic comparisons of proximity to industrial sites for cases and controls are presented in Table 2. The average annual distance from their residence to the nearest active industrial site from 1970 through 1997 was 7,745 m for cases and 8,540 m for controls; average distance to a site reporting air release of at least one chemical classified as at least a possible human carcinogen by IARC was also lower for cases, although neither difference approached statistical significance. A relatively small percentage of the population ever lived within half a mile of any site. Cases were more likely than controls to have resided within half a mile of any active site (10.1 versus 8.2%), any petrochemical site (7.9 versus 6.4%), any reported IARC possible, probable or known carcinogen releasing site (7.0 versus 5.9%), or any site releasing an IARC known carcinogen (5.9 versus 5.3%). The proportion of the population ever living within half a mile of a site was low, none of the observed differences reached statistical significance, and the difference between cases and controls narrowed rather than increased when going from any active site to those specifically reporting release of IARC-recognized carcinogens. Residence within one or two miles of a site was much more common than residence within half a mile, with nearly half of the study population ever resident within two miles of an active site. Since these comparisons do not address other critical potential risk factors, logistic regression models that adjust for smoking and other critical exposures are discussed next.

### 3.2. Models of Lung Cancer by Proximity to Industrial Sites Adjusted for Other Risk Factors

Table 3 presents the results of models adjusted for age, gender, race, education, metropolitan residence, and smoking history. The adjusted odds ratio for lung cancer was 1.05 (95% CI 0.59–1.86) for persons ever living within the half-mile buffer around any active site. Similar ORs were noted for residence within the half-mile buffer around any petrochemical site (1.10) or any IARC 1, 2A or 2B rated chemical releasing site (1.05), but not after restricting sites to those releasing known carcinogens (0.90). No elevated OR was noted when the buffer area was extended to one or two miles, regardless of the nature of the site. In fact, odds ratios at two miles showed a statistically significant reduction for IARC 1, 2A or 2B rated sites (0.69; 95% CI 0.48–0.99) as well as for IARC known carcinogen releasing sites (0.60; 95% CI 0.42–0.88). Results unadjusted for any other risk factors are also presented for reference. Most of the differences between the unadjusted and adjusted results are due to the addition of control for smoking. The odds ratio for residence within the half-mile buffer around a petrochemical plant, for example, drops from 1.26 to 1.07 with control for smoking and arrives at 1.10 with control for the remaining factors. 

To factor in duration as well as proximity, the results of models based on total years lived within the buffer are presented in Table 4. Persons who resided for at least 16 years within half a mile of any site had an adjusted lung cancer OR of 1.37 (0.63–2.96); this increased slightly to 1.45 (0.61–3.48) for those within a petrochemical site buffer, and fell to 1.25 (0.51–3.07) and 1.10 (0.42–0.92) for those within an IARC possible and IARC known carcinogen releasing site, respectively. When a separate category is added for 1–15 years' exposure, there is no indication of increasing odds of lung cancer across ascending exposure categories. Rather, the odds appear lower for 1–15 years' exposure compared to no exposure. No positive association is seen for years of residence in one- or two-mile buffers. The only statistically significant association is the lower OR seen for 1–15 years within the 2-mile buffers around an IARC known or possible carcinogen-releasing site, similar to the results for simply ever being within the buffer for one of these sites.

Further analyses were run to assess the potential effect of other factors on these results. Socioeconomic status in its various measures has been linked to lung cancer, so alternative measures were examined. Substitution of income for education in the regression models produced similar results; inclusion of both variables simultaneously had little effect beyond adding variance to the model. Addition of fruit and vegetable intake, self-reported occupational exposure to one or more potential lung carcinogens, or high risk occupation based on job history likewise produced no meaningful change in results. The same held for use of the square root of pack-years in place of the separate years smoked and cigarettes per day terms used in the main analyses, or exclusion of residences that had been geocoded to the zip code centroid rather than via exact street match. An index based on years within a buffer wherein weights of 2, 1, and 0.5 were assigned if the highest IARC carcinogen rating of any chemical reported released by the site was known, probable, or possible was also used. Results were similar to those for unweighted years in Table 4, although several of the odds ratios increased slightly. 

Analyses extending the lag period to 15 years were conducted. While many of the odds ratios became larger to a degree, the basic conclusions were similar to those reached with a 5-year lag: the strongest associations were seen for a 0.5 mile buffer, and none of them approached statistical significance. There was no consistent increase in odds with increasing years of exposure.

## 4. Discussion

This analytic study is generally consistent with the findings of two earlier ecologic studies of lung cancer and industrial proximity in Louisiana that found no statistically significantly elevated risk of lung cancer [12, 14]. Some associations were observed between residence near any industrial or any petrochemical site at a distance of half a mile or less and lung cancer, but these associations did not approach statistical significance, show evidence of increasing effect across increasing categories of cumulative exposure, or appear higher for proximity to plants that reported releases to air of possible, probable, or known human carcinogens according to current IARC criteria. 

Most previous studies of industrial site proximity and lung cancer have focused on smelters. Few studies have addressed petrochemical sites, and most of those employed an ecologic study design and did not control for competing individual level risk factors. Belli et al. [16] conducted a case-control study of cancer mortality around an Italian petrochemical plant. They noted a tripling of the lung cancer odds ratio when the longest-held residence was within 2 km of the plant after adjusting for smoking, occupation, and education. Despite the magnitude of the odds ratio, this association did not reach statistical significance, however, due to a paucity of lung cancer cases. More recently, Edwards et al. carried out a case-control study of female lung cancer around Teesside, England, which contains six heavy industrial works, including three petrochemical operations [17]. Two clusters of heavy industry were identified and three exposure zones were created within Teesside based on proximity to these clusters; residential histories were obtained and each residence was assigned to an exposure zone. An elevated odds ratio (1.82) was observed for more than 25 years compared to 0 years within the zone closest to industry, but not for 1–25 years. The elevation did not reach statistical significance. Adding a 20-year lag cut the odds ratio for 25 years of residence in half. Adding residences outside of Teesside with exposure assigned based on self-reported proximity to heavy industry produced a smaller odds ratio without the lag but a higher one with it. A previous study of Teesside based on recorded mortality rates had found elevated lung cancer SMRs for the area as a whole compared to England and Wales, although the SMR was higher in the most industrially exposed zone only for women, not for men [18].

The current study's findings thus fall in the range observed across these recent case-control studies and other previous studies of lung cancer around petrochemical sites. The observation that no evidence of any elevated odds of lung cancer was found among women differs from the findings in the studies of the Teesside population, but among other studies reporting relevant results Kaldor found an association only in men and Tsai in neither men nor women [10, 14].

Confounding due to potential effects of particulate matter or other pollutants derived from nonindustrial sources could have contributed to the lack of association noted between industrial site proximity and lung cancer. A number of studies in the U.S. and Europe have observed a positive association between such measures, particularly PM2.5, PM10, or TSP, and lung cancer [18–25]. Despite often large study populations, only in the latest extension of the complete ACS analyses [23] and the ASHMOG study [21] did these results reach statistical significance. In the current study, analyses restricted to metropolitan or nonurban areas, which should have reduced variability in background exposure to particulate or other exposure from non-industrial sites, still failed to yield consistent associations between industrial site proximity and lung cancer.

Strengths of the study include the restriction to histologically confirmed cancers (minimizing risk of misdiagnosis) and in-person interviews (avoiding potential inaccurate or biased recall of exposure history or personal habits introduced by use of proxy interviews). Thorough data on smoking, occupation, socioeconomic status, diet, and other potential risk factors was available and thus could be controlled for. This is critical as smoking, low socioeconomic status, and residence close to an industrial site tend to correlate, and failure to account for each factor can potentially leave results overrun with bias [26]. Our analyses bore this out: control for tobacco smoking and education reduced the positive odds ratios for site proximity measures seen in unadjusted results; the incremental effect of adding control for education after that for smoking was much less marked. Low socioeconomic status acts as a marker for many factors besides smoking, including poorer diets and occupations with greater exposure to potential carcinogens, but these risk factors convey much lower risk of lung cancer than smoking. Control for the latter factors did not materially alter our results, however—not surprising for occupational exposure, given that such exposures were much less common than smoking in our population and would generally not elevate individual risk as much. A 30-year residential history was collected rather than relying on most recent residence, and geocoding enabled direct computation of distance to each industrial site within the catchment region. This was augmented by site- and chemical-specific air release data.

The study was a population-based case-control study. While an analytical sample of 892 people with detailed data is substantial, less than 10% of the population held a residence within half a mile of any industrial site for even a single year, yielding limited power for assessment of associations with high-proximity exposures. Power is greater for residence within one or two-mile buffers, but it is unclear to what degree extending residential coverage to one or two miles from a site still represents an indicator of high exposure risk rather than of being at a “safer” distance from the site.

The potential for recall bias exists in any case-control study. Given that site proximity measures were obtained by mapping residential address information and calculating distances across that history to over 90 potential exposure sites rather than asking participants directly about their exposure, however, systematic recall bias is unlikely for the main exposure under study. Further, all smoking (and other) histories were obtained without use of proxies, reducing potential differences in data quality between cases and controls, and all interviews were conducted using standardized forms and techniques.

Case accrual in population-based studies of lung cancer presents special challenges due to short survival periods following diagnosis and debilitating illness. Even with rapid case ascertainment, nearly as many cases were deceased or too ill to interview as were enrolled. Lung cancer patients who died rapidly or were too ill to interview shortly after diagnosis were likely to have presented at later stage than those who stayed healthy long enough to participate. The implications of missing these more advanced cancers are unclear. It is possible that if exposure to petrochemical plant emissions promotes more aggressive tumors, these cases would be underrepresented in the study population. All but 54 eligible cases were able to be contacted, reducing any threat to the representativeness of the sample from this source.

Low response rates, particularly markedly lower rates among controls, heighten the potential for bias in results. With response rates of 73% among cases and 58% among controls, the latter is higher than typically achieved in field studies, reducing potential for bias. Demographic information regarding nonresponding controls was limited. Nonrespondents did tend to be older by around two years among cases and among controls, younger by around four years. Thus characteristics tracking with age should tend to be overrepresented in the control population, although the small magnitude of the age shift argues against a major impact. As a comparison, Edwards et al. [17] experienced a 48% response rate for their controls. With rich information available on nonresponders through physician registries, they found little evidence of systematic differences.

The TRI data used to classify sites according to the IARC carcinogen ratings of their reported releases of specific chemicals is a putative study strength. Detailed data were only available from 1988 or later, depending upon when the reporting of a particular chemical became mandatory. It is possible that some sites may have released IARC 1, 2A, or 2B rated chemicals only prior to this period and thus been misidentified as nonreleasers. Further, the actual amount of each chemical released was not addressed due to the difficulty in estimating differences in chemicals' carcinogenic potency. Both factors may have contributed to inaccurate potential dose and effect estimation. Further complicating estimation of dose is the potential contribution of wind direction and stack height on distribution of releases. The latter could in fact result in released chemicals passing over very near areas before settling at greater distances from the site. Differences in dispersion characteristics may combine with release height to affect dose (e.g., near-ground release of a volatile organic versus high air release of a particulate). The inability to incorporate stack height coupled with specific pollutant dispersion characteristics into the model could have had an influence on results.

The issue of latency was addressed to a degree by the incorporation of a 5-year lag in the main analyses. There was, however, no increase in the observed associations between site proximity and lung cancer when a 5-year lag was included compared to no lag. Most associations, in fact, grew somewhat weaker after adoption of the lag. A limitation of the study is that the available residential history is restricted to approximately 30 years. This makes exploration of longer lags problematic. A 15-year lag did yield stronger associations on average, but again even the largest of these did not approach statistical significance. Further, the longer lag sacrifices much of the available residential history, forcing reliance on a smaller timeframe as well as greater dependence on extrapolation beyond available chemical release data, which makes it unclear whether the observed differences support a longer latency effect or simply reflect random change produced by excluding some of the data. It is notable that a recent case-control study of petrochemical and other plant emissions similar in design to our own [17] found that incorporation of a 20-year lag produced changes of opposite direction depending on whether it was applied to their primary target or expanded study population, despite the availability of lifetime residential history.

## 5. Conclusions

The evidence linking environmental exposure to petrochemical plant releases with lung cancer is equivocal. Both ecologic and case-control studies have yielded mixed conclusions. The current case-control study found only small indications of an association between residential proximity to petrochemical or other industrial plants in the lower Mississippi river corridor and odds of lung cancer, with no evidence of dose-response observed. The study's findings do not support a statistically significant effect of petrochemical or other industrial sites releases on the risk of lung cancer among the general population. Larger sample sizes and more definitive measures of exposure would be needed to conclusively resolve whether any effects on risk actually exist, or whether any effect is observed in subgroups defined by genetically determined host response.

## Figures and Tables

**Table 1:** Demographic and smoking characteristics of the LMRICS study population^1^.

Descriptor	Total study population	Cases	Controls
Number	892	455	437
Age (mean, in years)	60.0 (10.2)	60.4 (9.2)	59.6 (11.1)
Male (%)	64.5	65.3	63.6
Black (%)	35.3	36.0	34.6
High school graduate or higher (%)	75.0	66.2	84.2
Residences (1970–99)—total number (mean)	2.98 (2.41)	2.89 (2.33)	3.07 (2.48)
Residences—total number geocodable (mean)	2.91 (2.35)	2.82 (2.26)	3.01 (2.41)
Current smokers (%)	44.4	64.6	23.3
Smoking-years (mean)	26.9 (18.7)	36.1 (14.4)	17.4 (17.9)
Cig/day while smoking (mean)	21.6 (21.6)	28.6 (17.6)	14.3 (17.9)

^1^Means are presented followed by standard deviations in parentheses as appropriate.

**Table 2:** Proximity to industrial sites for lung cancer cases and controls in the LMRICS study: mean distance to sites and distribution of ever residing within buffer around specific site types, 1970–1997^1^.

Site type	Cases	Controls
Average distance to any active site (m)	7745	8530
Average distance to IARC 1, 2A or 2B rated site^2^ (m)	9662	9952
Ever within 0.5 mi buffer, any active site	10.1%	8.2%
Ever within 1.0 mi buffer, any active site	22.0%	20.1%
Ever within 2.0 mi buffer, any active site	51.9%	47.1%
Ever within 0.5 mi buffer, any petrochemical site	7.9%	6.4%
Ever within 1.0 mi buffer, any petrochemical site	14.9%	15.8%
Ever within 2.0 mi buffer, any petrochemical site	39.8%	35.2%
Ever within 0.5 mi buffer, any IARC 1, 2A or 2B rated^2^ site	7.0%	5.9%
Ever within 1.0 mi buffer, any IARC 1, 2A or 2B rated^2^ site	12.7%	14.4%
Ever within 2.0 mi buffer, any IARC 1, 2A or 2B rated^2^ site	31.2%	32.0%
Ever within 0.5 mi buffer, any IARC 1 rated^3^ site	5.9%	5.3%
Ever within 1.0 mi buffer, any IARC 1 rated^3^ site	11.0%	12.8%
Ever within 2.0 mi buffer, any IARC 1 rated^3^ site	26.2%	29.1%

^1^None of the differences between cases and controls approached nominal statistical significance (*P* < .05).

^2^Site reporting release of at least one chemical rated as a known, probable, or possible human carcinogen by IARC.

^3^Site reporting release of at least one chemical rated as a known human carcinogen by IARC.

**Table 3:** Ever Living within Buffer around Specific Site Types, Lung Cancer Crude and Adjusted Odds Ratios with 95% Confidence Intervals^1^.

Site type		Buffer extension	
	0.5 mile	1 mile	2 mile
*Adjusted results*			
Any active site	1.05	0.86	0.97
	(0.59–1.86)	(0.57–1.28)	(0.70–1.34)
Any petrochemical site	1.10	0.73	0.95
	(0.58–2.08)	(0.46–1.15)	(0.68–1.34)
Any IARC 1, 2A or 2B rated site	1.05	0.70	0.69
	(0.53–2.06)	(0.43–1.14)	(0.48–0.99)
Any IARC 1 rated site	0.90	0.64	0.60
	(0.44–1.85)	(0.38–1.07)	(0.42–0.88)
*Unadjusted results*			
Any active site	1.25	1.12	1.21
	(0.79–1.98)	(0.81–1.54)	(0.93–1.57)
Any petrochemical site	1.26	0.94	1.21
	(0.75–2.10)	(0.65–1.35)	(0.93–1.59)
Any IARC 1, 2A or 2B rated site	1.20	0.87	0.96
	(0.70–2.04)	(0.59–1.27)	(0.73–1.28)
Any IARC 1 rated site	1.14	0.84	0.87
	(0.64–2.01)	(0.56–1.26)	(0.64–1.16)

^1^All odds ratios are for persons ever living within buffer of specified extension around a specified type of site. Adjusted results include control for age, gender, race (Caucasian non-Hispanic: yes versus no), current smoker status (yes/no), years smoked, average cigarettes/day if and when smoking, education, and residence within New Orleans metropolitan area.

**Table 4:** Adjusted odds of lung cancer for increasing categories of years lived within the buffer around specific site types, with 95% confidence intervals in parentheses^1^.

Site type	Years within buffer: 16 + versus under 16 years		Years within buffer: 16 + or 1–15 versus 0 years		
	0–15	16 or more	0	1–15	16 or more
*0.5 mile Buffer*					
Any active site	1.00	1.37 (0.63–2.96)	1.00	0.68 (0.31–1.49)	1.35 (0.62–2.91)
Any petrochemical site	1.00	1.45 (0.61–3.48)	1.00	0.68 (0.29–1.64)	1.43 (0.60–3.44)
Any IARC 1, 2A or 2B rated site	1.00	1.25 (0.51–3.07)	1.00	0.68 (0.27–1.74)	1.24 (0.50–3.04)
Any IARC 1 rated site	1.00	1.10 (0.42–0.92)	1.00	0.61 (0.23–1.62)	1.09 (0.41–2.89)
*1.0 mile buffer*					
Any active site	1.00	0.89 (0.53–1.50)	1.00	0.81 (0.48–1.37)	0.87 (0.51–1.47)
Any petrochemical site	1.00	0.70 (0.38–1.31)	1.00	0.76 (0.41–1.41)	0.66 (0.37–1.17)
Any IARC 1, 2A or 2B rated site	1.00	0.68 (0.37–1.24)	1.00	0.68 (0.35–1.35)	0.66 (0.36–1.21)
Any IARC 1 rated site	1.00	0.64 (0.34–1.21)	1.00	0.63 (0.31–1.27)	0.62 (0.33–1.18)
*2.0 mile buffer*					
Any active site	1.00	1.05 (0.75–1.48)	1.00	0.86 (0.54–1.36)	1.02 (0.71–1.46)
Any petrochemical site	1.00	0.96 (0.68–1.48)	1.00	0.88 (0.54–1.44)	0.94 (0.64–1.38)
Any IARC 1, 2A or 2B rated site	1.00	0.80 (0.52–1.23)	1.00	0.55 (0.32–0.93)	0.74 (0.50–1.10)
Any IARC 1 rated site	1.00	0.84 (0.54–1.30)	1.00	0.42 (0.24–0.72)	0.72 (0.47–1.11)

^1^All odds ratios are for persons living for a given number of years within the buffer of specified extension around a specified type of site, adjusted for: age, gender, race (Caucasian non-Hispanic: yes versus no), current smoker status (yes/no), years smoked, average cigarettes/day if and when smoking, and education (less than high school, high school, post-high school).

**Table 5:** 

SIC Code	Type of industry
2865	Cyclic crudes and intermediates
2869	Industrial organic chemicals NEC
5169	Chemicals and allied products NEC
2812	Alkalies and chlorine
2899	Chemical preparations NEC
2911	Petroleum refining
2821	Plastics materials and resins
2851	Paints and allied products
2822	Synthetic rubber

## Appendix

### A. Criteria Used to Classify Industrial Sites as a Petrochemical Site

Standard Industrial Classification codes for all industrial sites served as the basis for determining whether these should be classified as petrochemical sites for purposes of the study analyses. Sites with any of the SIC codes listed in Table 5 were classified as a petrochemical site.

The codes for 5171 (PETROLEUM BULK) and 2824 (ORGANIC FIBERS) were not included among those classified as petrochemical sites due to their low potential for exposure of off-site populations to carcinogenic chemical release. For facilities with code 5169 (CHEMICALS AND ALLIED PRODUCTS NEC), TRI chemical release data for that facility were checked and if no releases of chemicals rated 1, 2A, or 2B by IARC were reported in the available data, the facility was not classified as a petrochemical site. The same approach was taken for codes of 2851 (PAINTS AND ALLIED PRODUCTS).
